# Leukocyte recruitment in preterm and term infants

**DOI:** 10.1186/s40348-016-0063-5

**Published:** 2016-10-24

**Authors:** Katinka Karenberg, Hannes Hudalla, David Frommhold

**Affiliations:** 1 Department of Neonatology, University Children’s Hospital, Im Neuenheimer Feld 430, 69120 Heidelberg, Germany; 2 Department of Newborn Medicine, Brigham and Women’s Hospital, Harvard Medical School, Boston, MA USA

**Keywords:** Leukocyte recruitment, Preterm, Newborn, Fetus, Rolling, Adhesion, Transmigration, Neutrophil, Sepsis, Innate immunity

## Abstract

Impaired cellular innate immune defense accounts for susceptibility to sepsis and its high morbidity and mortality in preterm infants. Leukocyte recruitment is an integral part of the cellular immune response and follows a well-defined cascade of events from rolling of leukocytes along the endothelium to firm adhesion and finally transmigration which is concerted by a variety of adhesion molecules. Recent analytical advances such as fetal intravital microscopy have granted new insights into ontogenetic regulation and maturation of fetal immune cell recruitment. Understanding the fetal innate immune system is essential for targeted prevention and therapy of premature infants with severe infections or disorders of the immune system. This review gives an overview of the basic principles of leukocyte recruitment, particularly neutrophil trafficking, and its development during early life and highlights technical limitations to our current knowledge.

## Introduction

Prematurity is the most prominent risk factor for neonatal diseases and death [1]. Despite medical progress in newborn medicine, mortality remains high since the number of very low birth weight infants (≤1500 g) increases globally [2–4]. Especially among very immature infants, infection and sepsis are still the leading causes for mortality and morbidity [2, 5]. This may in part be explained by the immaturity of the innate immune system, which preterm infants (<37 weeks of gestation) heavily rely on as the adaptive immune system is not yet formed [6]. Intrauterine fetal immunosuppression plays a key role in preventing excessive adverse immune reactions at the feto-maternal placental border. Yet, this beneficial intrauterine feature proves disadvantageous in preterm infants lacking maternal immune protection while being exposed to high levels of pathogens. Up to 60 % of extremely premature infants (<28 weeks of gestation and/or <1000 g birth weight) may suffer from bacterial sepsis in contrast to less than 5 % of late preterm and term neonates [7], which indicates that the immune response evolves throughout gestation. The mortality risk sharply decreases with each additional week of gestation and rise in birth weight [4]. Researchers are only beginning to understand the complex ontogenetically regulated maturation of the fetal immune system and how to alter or support this transition.

The impaired function of the premature immune system has multiple causes: lack of immunoglobulins [8] and antimicrobial peptides [9], low levels of circulating complement factors, and lack in total number and maturation of immune cells [10]. Neutropenia and immature neutrophil trafficking partially account for the high susceptibility to opportunistic and bacterial infections [11]. Despite increasing evidence for a highly complex role of leukocytes in both innate and adaptive immunity, this short review focuses on the ontogenetic development of leukocyte recruitment, in particular polymorphonuclear neutrophils (PMN), as one key component of innate immunity in preterm neonates.

## Leukocyte recruitment

**Fig. 1:**
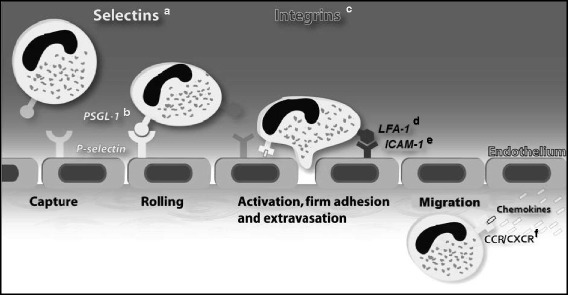
Leukocyte recruitment cascade. The multistep recruitment cascade is illustrated using the example of a neutrophil. It starts with the capture of circulating neutrophils from the blood stream, followed by selectin-dependent rolling and chemokine- and integrin-dependent adhesion. After extravasation, the neutrophil follows chemokine gradients through the tissue to the point of damage/inflammation. Developmental alterations of adhesion molecule expression are indicated by footnotes as follows (fetal/premature levels were compared to those of adults): (*a*) L-selectin - reduced [26, 33, 37, 45, 46] and unchanged [47], E-selectin - reduced [29, 31, 38], P-selectin - reduced [29, 34, 38, 39]; (*b*) PSGL-1 - reduced [29, 31, 36]; (*c*) Mac-1 - reduced [26, 31, 36], unchanged [30, 33], and increased [37]; (*d*) LFA-1 - reduced [26, 45] and unchanged [30, 31, 36, 37]; (*e*) ICAM-1 - reduced [29, 31, 38]; (*f*) CXCR2 - unchanged [31]

Leukocyte recruitment is an integral part of the cellular immune response and follows a defined cascade of events [12]. After recognition of invading pathogens, leukocytes are stimulated with the primary purpose of eliminating the inflammatory source. This multistep process starts with the capture of circulating leukocytes from the blood stream, mostly in postcapillary venules in close proximity to inflamed tissue. A simplified version of the leukocyte recruitment cascade exemplified for neutrophils is displayed in Fig. 1 (developmental aspects are indicated by footnotes and discussed in the next chapter).

The initial step of leukocyte rolling is mediated by selectins, which bind to their respective ligands such as P-selectin glycoprotein ligand 1 (PSGL-1), CD44, or E-selectin ligand 1 (ESL-1) [13, 14]. The three known members of the selectin family are L-, P-, and E-selectin. After activation of endothelial cells, adhesion molecule upregulation drives leukocyte adhesion to the vessel wall. The interaction of selectins and chemokines with their respective receptors triggers integrin activation (e.g. macrophage antigen 1 (Mac-1), lymphocyte function-associated antigen 1 (LFA-1)) which in turn bind to their endothelial ligands (intercellular adhesion molecule 1 (ICAM-1), vascular cell adhesion molecule 1 (VCAM-1), receptor for advanced glycation endproducts (RAGE)) [15] leading to leukocyte deceleration and finally adhesion [16]. The firm leukocyte adhesion is crucially mediated by tight bonds between integrins and their ligands balanced by permanent inside-out integrin signaling (intracellular signaling activates integrin on cell-surface) and outside-in integrin signaling (ligand-induced activation of intracellular signaling pathways) [12, 17–19]. Subsequently, spreading is induced by integrin-mediated rearrangement of actin cytoskeleton followed by crawling along the endothelium in order to find a designated site to transmigrate from vessel into inflamed tissue [20, 21]. Intravascular chemokine gradients guide leukocytes to sites of damage [22]. Transmigration depends on many different factors like distribution and density of integrin ligands, chemoattractants, and other cytokines as well as adhesive ligands [23]. Two different routes of transmigration are known: paracellular at endothelial borders (70–90 %) [14] or transcellular. After transendothelial cell migration (TEM), leukocytes display an altered phenotype, enhanced survival, and enhanced ability to eliminate pathogens [13]. For sufficient diapedesis, a weakening of endothelial junctions and an increase in cytosolic free calcium is required [24]. The site of transmigration may depend on the condition of junctions, so leukocytes are likely to take the nearest route with least resistance in an acceptable range. The key molecules involved in the leukocyte recruitment cascade are summarized in Table 1 based on a recent review by Vestweber [25].

**Table 1:** Leukocyte adhesion molecules

Endothelial adhesion molecule	Leukocyte ligand	Endothelial ligand	Functions
E-selectin	PSGL-1, CD44, ESL-1	None	Capturing, rolling, integrin activation
P-selectin	PSGL-1	None	Capturing, rolling, integrin activation
ICAM-1	LFA-1, Mac-1	None	Rolling, adhesion, crawling
VCAM-1	VLA4	None	Rolling, adhesion, crawling
RAGE	Mac-1	None	Adhesion, crawling, transmigration
ICAM-2	LFA-1, Mac-1	None	Crawling, initiating diapedesis
JAM-A	LFA-1	JAM-A	Leukocyte diapedesis
JAM-B	VLA-4	JAM-B, JAM-C	Prevention of reverse TEM
JAM-C	Mac-1	JAM-C, JAM-B	Prevention of reverse TEM
ESAM	Unknown	ESAM	Diapedesis
PECAM-1	PECAM-1	PECAM-1	Promoting TEM
CD99	CD99	CD99	Promoting TEM
CD99L2	CD99L2	CD99L2	Promoting TEM
VE-cadherin	None	VE-cadherin	Preventing diapedesis

Location and function of key leukocyte adhesion molecules and their ligands [15, 25, 48]

*ESAM* endothelial cell-selective adhesion molecule, *CD99L2* CD99 antigen-like protein 2, *JAM* junctional adhesion molecule, *PECAM*-*1* platelet endothelial cell adhesion molecule 1, *VE*-*cadherin* vascular endothelial cadherin, *VLA4* very late antigen 4, *TEM* transendothelial migration

## Maturation of fetal leukocyte recruitment

Understanding the fetal innate immune system is essential for targeted prevention and therapy of premature infants with severe infections or disorders of the immune system. The high vulnerability of preterm neonates to suffer from severe infections and sepsis can partially be attributed to impaired leukocyte recruitment early during fetal life [11]. The observation of reduced fetal leukocyte trafficking and chemotaxis is mainly explained by diminished expression of leukocyte adhesion molecules and production of cytokines at this developmental stage [26–28]. Expression profiles of the most relevant leukocyte adhesion molecules during fetal life is compared to that of adults in Table 2 and also depicted in Fig. 1 by respective footnotes.

**Table 2:** Expression of leukocyte adhesion molecules in neonates and adults

Molecule	Cell type	Expression in fetuses/premature neonates compared to adults and respective references	
Mac-1	PMN	↓ Reduced ↑ Increased ↔ Equal	[26, 31, 36] [37] [30, 33]
LFA-1	PMN	↓ Reduced ↔ Equal	[26, 45] [30, 31, 36, 37]
CXCR2	PMN	↔ Equal	[31]
CD 18	PMN	↓ Reduced	[26]
L-selectin	PMN	↓ Reduced ↔ Equal	[26, 33, 37, 45, 46] [47]
E-selectin	Skin EC Yolk sac vessels	↓ Reduced ↓ Reduced ↓ Reduced	[38] [31] [29]
P-selectin	EC Fetal skin PMN Yolk sac vessels	↓ Reduced ↓ Reduced ↓ Reduced ↓ Reduced	[34] [38] [39] [29]
RAGE	PMN	↑ Increased	[30]
ICAM-1	Skin EC Yolk sac vessels	↓ Reduced ↓ Reduced ↓ Reduced	[38] [31] [29]
ICAM-2	Yolk sac vessels	↓ Reduced	[29]
VCAM-1	Skin Yolk sac vessels	↓ Reduced ↓ Reduced	[38] [29]
PSGL-1	PMN Yolk sac vessels	↓ Reduced ↓ Reduced	[31, 39] [29]
PECAM-1	EC Yolk sac vessels	↔ Equal ↔ Equal	[31] [29]
VE-cadherin	EC	↔ Equal	[31]
VLA-4	PMN	↓ Reduced	[45]

*PECAM*-*1* platelet endothelial cell adhesion molecule 1, *VE*-*cadherin* vascular endothelial cadherin, *VLA4* very late antigen 4, *EC* endothelial cells

Sperandio et al. showed in vivo that neutrophil rolling and adhesion in murine yolk sac vessels is strongly reduced at early gestational ages and increases throughout gestation [29]. These observations were validated in human preterm and term leukocytes in vitro using flow chamber experiments as a rule-in/rule-out approach [30]. In the same experimental setting, it has been shown that RAGE, a key metabolic receptor of diabetic patients, controls neutrophil adhesion in preterm and term infants [30].

Postnatal maturation of immune response and leukocyte recruitment is driven by multiple new environmental factors [31]. Several studies describe significantly reduced neutrophil transmigration and chemotaxis in neonates compared to adults [11, 29, 32]. Notably, cellular immunity of preterm infants matures slower and later than in term infants [11]. Expression of L-, P-, and E-selectin was reported to be reduced in mature neonates in vitro and in vivo compared to infants and adults [31, 33, 34]. On the other hand, posttranslational glycosylation of selectin ligands is augmented during the neonatal period [35]. In most studies, fetal expression of integrins such as Mac-1 and LFA-1 were described to be reduced or equal compared to adults [26, 30, 31, 33, 36, 37]. Similar observations have been described for other adhesion molecules such as ICAM-1/-2, VCAM-1, VLA4, and PSGL-1 [29, 31, 38, 39]. Although fetal expression of the majority of adhesion molecules is low when compared to term infants and adults [11, 26, 36], others, like CXCR2, PECAM-1, and VE-cadherin (and LFA-1), are equally expressed in premature and mature neonates and adults (Table 2) [29, 31, 37].

Taken together, the gestational age-dependent upregulation of adhesion molecules leads to functional maturation of leukocyte rolling, adhesion, transmigration, and chemotaxis, which in turn strengthens the innate immune response.

## Analytical limitations and outlook

Despite technical progress, human neonatal in vivo imaging of immune cell trafficking is not yet available. Thus, fetal leukocyte recruitment has mainly been studied in vitro using leukocytes and endothelial cells in dynamic flow chambers or transmigration assays [15, 30, 31, 40, 41]. Reports about in vivo investigations of fetal leukocyte recruitment in animals are limited and were mostly performed in nonmammalian organisms [42, 43]. A recently, developed intravital microscopic mouse model now offers the chance to visualize rolling and adhesive behavior of leukocytes during different stages of fetal development (E13–18) in vivo [29]. However, its microsurgery and microscopic approach is technically challenging and introduction of clinically relevant conditions difficult (Hudalla et al. in preparation). Moreover, the exploration of underlying mechanisms is often limited by sample sizes [5, 23, 24].

While our understanding of the fetal and early neonatal immune system is ever growing, treatment options are still limited and the vast majority of pharmaceutical trials are run in adults with fully developed immunity. Novel analytical tools and models to study innate immunity may facilitate the development of new gestational age- and sepsis stage-specific therapeutic approaches to fine-tune the premature immune system and thereby optimize the treatment of neonatal infections and sepsis [44].

## Abbreviations

CD99L2: CD99 antigen-like protein 2
EC: Endothelial cell
ER: Endoplasmic reticulum
ESAM: Endothelial cell-selective adhesion molecule
ESL1: E-selectin ligand 1
fMLP: N-Formylmethionyl-leucyl-phenylalanine
ICAM: Intercellular adhesion molecule
JAM: Junctional adhesion molecule
LFA-1: Lymphocyte function-associated antigen 1
LPS: Lipopolysaccharide
Mac-1: Macrophage antigen 1
PECAM-1: Platelet endothelial cell adhesion molecule 1
PMN: Polymorphonuclear neutrophils
PSGL-1: P-selectin glycoprotein ligand 1
RAGE: Receptor for advanced glycation endproducts
rtPCR: Real-time polymerase chain reaction
TEM: Transendothelial migration
VAP1: Vascular adhesion protein 1
VCAM-1: Vascular cell adhesion molecule 1
VE-cadherin: Vascular endothelial cadherin
VLA4: Very late antigen 4
